# Research on a New Method of Macro–Micro Platform Linkage Processing for Large-Format Laser Precision Machining

**DOI:** 10.3390/mi16020177

**Published:** 2025-01-31

**Authors:** Longjie Xiong, Haifeng Ma, Zheng Sun, Xintian Wang, Yukui Cai, Qinghua Song, Zhanqiang Liu

**Affiliations:** 1 School of Mechanical Engineering, Shandong University, Jinan 250061, China; 202214332@mail.sdu.edu.cn (L.X.); caiyukui@sdu.edu.cn (Y.C.); ssinghua@sdu.edu.cn (Q.S.); melius@sdu.edu.cn (Z.L.); 2 State Key Laboratory of Advanced Equipment and Technology for Metal Forming, Shandong University, Jinan 250061, China; 3 Key Laboratory of High Efficiency and Clean Mechanical Manufacture of Ministry of Education, Jinan 250061, China; 4 Key National Demonstration Center for Experimental Mechanical Engineering Education, Jinan 250061, China; 5 State Key Laboratory for Manufacturing Systems Engineering, Xi’an Jiaotong University, No. 28, Xianning West Road, Xi’an 710049, China; zheng.sun@xjtu.edu.cn; 6 Xi′an Modern Chemistry Research Institute, Xi′an 710065, China; wangxintian1@stu.xjtu.edu.cn; 7 School of Mechanical, Electrical & Information Engineering, Shandong University, Weihai 264209, China

**Keywords:** laser processing, linkage processing, macro–micro structure, galvanometer scanner, trajectory distribution, optimal time

## Abstract

In recent years, the macro–micro structure (servo platform for macro motion and galvanometer for micro motion) composed of a galvanometer and servo platform has been gradually applied to laser processing in order to address the increasing demand for high-speed, high-precision, and large-format precision machining. The research in this field has evolved from step-and-scan methods to linkage processing methods. Nevertheless, the existing linkage processing methods cannot make full use of the field-of-view (FOV) of the galvanometer. In terms of motion distribution, the existing methods are not suitable for continuous micro segments and generate the problem that the distribution parameter can only be obtained through experience or multiple experiments. In this research, a new laser linkage processing method for global trajectory smoothing of densely discretized paths is proposed. The proposed method can generate a smooth trajectory of the servo platform with bounded acceleration by the finite impulse response (FIR) filter under the global blending error constrained by the galvanometer FOV. Moreover, the trajectory of the galvanometer is generated by vector subtraction, and the motion distribution of macro–micro structure is accurately realized. Experimental verification is carried out on an experimental platform composed of a three-axis servo platform, a galvanometer, and a laser. Simulation experiment results indicate that the processing efficiency of the proposed method is improved by 79% compared with the servo platform processing only and 55% compared with the previous linkage processing method. Furthermore, the method can be successfully utilized on experimental platforms with good tracking performance. In summary, the proposed method adeptly balances efficiency and quality, rendering it particularly suitable for laser precision machining applications.

## 1. Introduction

Laser processing is widely utilized in industrial processing owing to its advantages of non-contact [1], high flexibility [2], high efficiency [3,4], and high processing accuracy [5,6]. In recent years, the demand for large-format, high-speed, and high-precision laser precision processing has been increasing [7,8,9], such as laser marking [10] for electronic devices, laser polishing [11], laser cutting [12], laser lithography, and so forth. There have been efforts using adaptive optics [13,14] to control the laser beam to expand the size of the laser spot at the focus to increase the processing area, but this way only expands the processing area at the focus and cannot effectively expand the overall processing area. The existing research focuses on the combination of the servo platform and galvanometer to form the macro–micro structure for processing. The term “macro–micro” in this context specifically emphasizes the dynamic coupling between two distinct orders of actuators, designed to differentiate motion structures in terms of motion scale, precision, and dynamic response characteristics. Herein, the servo platform is responsible for macro motion to provide a large range of motion, and the galvanometer is responsible for micro motion, that is, a small range of fine motion with high precision and rapid response. The galvanometer can control the position of the laser spot quickly and accurately by swinging two orthogonal parabolic mirrors in a certain angle range. It has the characteristics of high scanning speed and fast response speed, but the processing range is constrained by the field-of-view (FOV) [15,16]. The servo platform is adopted to expand the processing area, combining the advantages of the two sub-motion systems to achieve continuous large-format precision processing.

In some studies, the macro–micro structure mentioned above has been identified as kinematically redundant mechanisms [17], which utilize redundant degrees of freedom to carry out subtasks while finishing the primary task to realize large-format precision processing. The traditional method based on the macro–micro structure is the step-and-scan method [18,19]. The pattern to be processed is divided into several small range processing areas first, and then the servo platform moves to the center of the first processing area and stops. Subsequently, the galvanometer begins to process this area. After the galvanometer has processed a single processing area, the servo platform moves to the center of the next processing area and repeats the cycle until all the processing areas are finished. The servo platform’s and the galvanometer’s repetitive start-stop motion results in low processing efficiency and poor surface quality at the boundary of the scanning areas, such as stitching errors and overburn.

The step-and-scan method, in terms of the motion of macro and micro structures, is merely a process of multi-device collaborative motion method. From the perspective of the control system, the macro and micro structures are independent of each other, which inevitably leads to stitching errors and other defects. To overcome this problem, a linkage processing concept, also referred to as the on-the-fly processing method, was initiated, which primarily realized the simultaneous motion of the servo platform and galvanometer under the same control system [20,21,22,23]. The key point of this method is to smoothen the servo platform’s motion by acceleration and deceleration planning under the constraint of the FOV of the galvanometer and generate the trajectory of the galvanometer through motion distribution to complete the high-frequency motion, such as sharp corners. Currently, only a limited number of researchers have carried out related works about the linkage processing of macro–micro structure composed of a galvanometer and the servo platform. Liu et al. [20] used three times finite impulse response (FIR) filtering to achieve the velocity planning of composite motion and macro platform and calculated the micro platform’s velocity planning by subtracting the velocity of composite motion and macro platform. Nevertheless, this method only achieves local smoothing for corners between two adjacent trajectory segments. In fact, time optimization for macro platforms can be further advanced to accomplish global smoothing of corners. Zhu et al. [21] generated the path for the servo platform by scaling the pattern composed of long segments through selecting new points on the angle bisectors of adjacent long segments. For certain patterns composed of multiple micro segments, such as a discretized butterfly pattern made up of B-spline curves, this method cannot correctly scale the target path. In other words, this method is not suitable for motion planning of such patterns composed of multiple micro segments. Considering the processing characteristics of the servo platform and galvanometer, Cui et al. [22] proposed a synchronized control method based on motion distribution using a moving average low-pass filter. In the same way, Wang et al. [23] decomposed the high-frequency and low-frequency components of the target trajectory and proposed a trajectory distribution filtering algorithm. However, the cutoff frequency of the filter they used for motion distribution was determined based on experience and multiple experiments, rather than derived from theoretical calculations. In summary, although the existing linkage processing methods eliminate the stitching errors caused by the step-and-scan method, there are still several unresolved issues associated with these methods. It is evident that existing methods overlook the potential advantages of the galvanometer’s FOV. By using local blending at corners, these methods allow the galvanometer to move at maximum range only at the start and end points of each trajectory segment, failing to fully utilize its FOV. In fact, the galvanometer can achieve close to the maximum range movement in the start, middle, and end of each trajectory segment, further optimizing the total processing time. In addition, the method of scaling the target trajectory based on the angle bisector to generate the servo platform’s motion trajectory is only suitable for multiple long segments and does not consider the target trajectory, such as the butterfly pattern. Along with this, some existing methods have the problem that the key distribution parameter of the filter must be determined by repeated experiments, which will increase the overall processing time.

In this paper, a laser linkage processing method based on the macro–micro structure composed of a galvanometer and servo platform is investigated for large-format, high-speed, and precision processing, which is suitable for complex patterns in practical machining. The proposed method demonstrates three key advancements: (1) The pre-discretization algorithm significantly enhances the method’s general applicability to diverse patterns. (2) The global blending error control algorithm maximizes galvanometer kinematic performance and improves processing efficiency. (3) This work establishes a new framework for motion decomposition and planning in large-small format structures. The method is described in detail below: A smooth trajectory generation algorithm for a servo platform with bounded acceleration based on an FIR filter is proposed. In order to ensure the universality of the method, a complex pattern like a butterfly composed of B-spline curves is discretized into multiple micro segments. At the same time, an error formula is established that takes into account the impact of several trajectories before and after a single corner, and the error of the servo platform trajectory is constrained within the FOV of the galvanometer by adjusting the velocity adjustment scaling factor. After interpolation, each interpolation point of the servo platform is mapped to the target trajectory in equal proportion, and the real-time position of the galvanometer is obtained by vector subtraction of the composite motion and the servo platform motion. Therefore, the motion distribution of the macro–micro structures is realized accurately. Subsequently, the feasibility of the method is verified through simulations and experiments conducted on a constructed experimental platform. The proposed method, the servo platform processing only, and the previous linkage processing method [20] are carried out, respectively, under the same pattern and experimental condition, and the advantages of the proposed method are verified in terms of efficiency and accuracy.

## 2. Laser Linkage Processing Method

**Figure 1:**
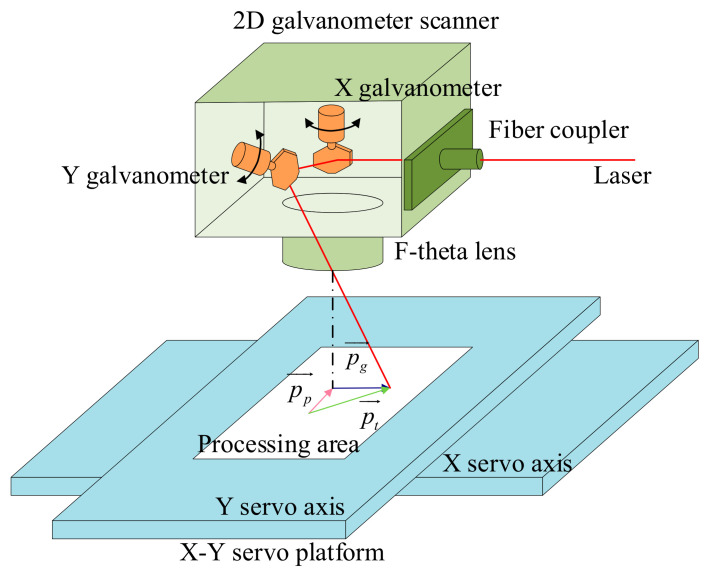
The schematic diagram of laser linkage processing method.

Figure 1 shows a classic laser linkage processing system, which consists of a two-axis servo platform (macro structure), a galvanometer scanner (micro structure), a laser generator, and other auxiliary components. The focal length of the F-theta lens restricts the working area of the galvanometer. When the focal length increases, the working area of the galvanometer (i.e., the FOV) widens, but the resolution decreases.

### 2.1. Criteria for Motion Planning

To fully use the large motion range of the servo platform and the galvanometer’s high-speed scanning, considering the different characteristics of the two sub-motion systems, a motion planning algorithm is designed. In the process of motion planning, the rules should be followed as follows:

1. The motion ranges of the galvanometer in the X and Y directions are within the FOV of the galvanometer and smaller than that of the servo platform, namely the following:

(1) $$\left\{\begin{array}{l} x_{g}\leq x_{\mathrm{FOV}}<x_{p}\\ y_{g}\leq y_{\mathrm{FOV}}<y_{p} \end{array}\right.$$

where *x_g_* and *y_g_*, respectively, represent the displacements of galvanometer in the X and Y directions, *x*_FOV_ and *y*_FOV_, respectively, represent the ranges of FOV in the X and Y directions, and *x_p_* and *y_p_*, respectively, represent the displacements of servo platform in the X and Y directions.

2. The speed and acceleration of the single axis of the servo platform should be within the allowable range of the servo drive, and the acceleration should be as small as possible to ensure the motion performance of the servo platform, which can be expressed as follows:

(2) $$\left\{\begin{array}{l} \left|a_{p}\right|\leq a_{\max}\\ \left|v_{p}\right|<v_{\max} \end{array}\right.$$

where *a_p_* and *a*_max_ are the acceleration and maximum acceleration values of the servo platform and *v_p_* and *v*_max_ are the speed and maximum speed values of the servo platform, respectively.

3. Under the constraints of rules 1 and 2, the motion planning of the servo platform should be as time-optimized as possible to improve the processing efficiency.

4. The motion of the servo platform and galvanometer is coupled, so for any time period *dt* in the machining process, the target displacement vector $\vec{p_{t}}$ is the sum of the servo platform displacement vector $\vec{p_{p}}$ and the galvanometer displacement vector $\vec{p_{g}}$ written as follows:

(3) $$\vec{p_{t}}=\vec{p_{p}}+\vec{p_{g}}$$

### 2.2. Description of Proposed Method

**Figure 2:**
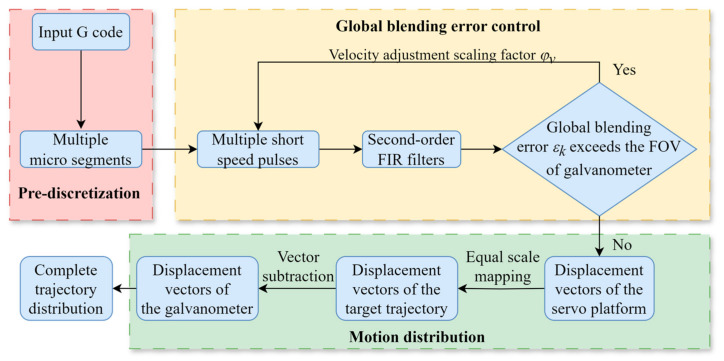
The flowchart of proposed laser linkage processing method.

Based on the motion planning criterion, the proposed laser linkage processing method in this paper contains three modules: pre-discretization, global blending error control, and motion distribution, as shown in Figure 2. In order to eliminate the limitation that only applies to long line segments like [21], this paper conducts research on the basis of continuous micro segments.

The initial G-codes are judged to mark the long G-codes among them, and they are pre-discretized into multiple micro G-codes. The coordinates of the micro segments after discretization can be obtained by Equation (10). Subsequently, the short speed pulses of the multiple micro segments are filtered twice with the FIR filter, whose delay time is determined by the kinematic performance of the servo axis, and the global blending error is derived under the constraint of the galvanometer’s FOV. The global blending error *ε_k_* can be calculated by Equation (16). In this paper, a velocity adjustment scaling factor *ϕ_v_* is proposed, and *ϕ_v_* can be calculated by Equation (21). If the error exceeds the limits of the FOV of the galvanometer, the initial speed pulse is adjusted by the *ϕ_v_* to keep the global blending error within the constraints, ensuring that the contour error generated by the servo platform is within the FOV of the galvanometer. After obtaining the displacement vectors of the servo platform, the displacement vectors of the target trajectory are calculated by the method of equal scale mapping, and the displacement vectors of the galvanometer can be obtained by vector subtraction from Equation (26). This method not only ensures that the synthetic motion of the galvanometer and the servo platform is the target trajectory but also ensures the time synchronization of the galvanometer and the servo platform.

### 2.3. Generation of an S-Shaped Velocity Curve Based on an FIR Filter

In order to make the motion stable with no impact shock and reduce the calculation time, the proposed method adopts FIR filter twice filtering to realize the S-shaped curve planning of the servo platform and ensures the continuous speed and acceleration, and the jerk is bounded. Furthermore, the motion planning method based on FIR filter does not need to determine the corner path first and then plan the corner velocity as in the previous method [19]. The proposed method only needs to automatically plan the corner path and corner velocity according to the corner error constraint, which improves the computational efficiency. The following is how single FIR filtering works:

The FIR filter *f_i_*(*t*) is defined in the real number domain, which is a rectangular pulse with a unit area, as shown in the following Equation (4):

(4) $$f_{i}(t)=\left\{\begin{array}{cc} 1/T_{i} & \;0\leq t\leq T_{i}\\ 0 & \;t<0\cup t>T_{i} \end{array}\right.$$

where *i* denotes the *i*th filter, *i* = 1, 2, and *T_i_* is the delay time of the filter *f_i_*(*t*), which is an integer multiple of the interpolation period *t_c_*, that is, *M*∈N*T_i_ = Mt_c_, *M*∈N*.

There is an interpolation point velocity sequence *x*(*t*) of synthetic motion; *t* represents the interpolation time point, and the value of *t* starts from 0 and increases according to the integer multiple of the interpolation period to the total motion time *t_t_* of this segment of the trajectory. *x*(*t*) is regarded as the input signal, which is filtered by Equation (5); the operation process is equivalent to the convolution operation between input signal *x*(*t*) and filter *f_i_*(*t*):

(5) $$\begin{array}{cc} v(t) & =f_{i}(t)*x(t)\\ & =\sum_{n=0}^{M-1}f_{i}(nt_{c})x(t-nt_{c}) \end{array}$$

where *v*(*t*) is the filtered velocity sequence of synthetic motion. Due to the unavoidable delay characteristic of the FIR filter, the total time of *v*(*t*) after filtering is increased to *t_t_* + *T_i_*.

In laser linkage processing, it is assumed that the target pattern is composed of *E* points, which are represented by a series of Cartesian coordinates **P**_k_ = [*P_x_*_,*k*_,*P_y_*_,*k*_]^T^, *k* = 1, 2, …, *E*∈N^*^, and each linear (G01) movement is realized by a set of tangential feed pulse commands *F_k_*. The speed pulse width *T_v_*_,*k*_ is determined by the length of the line segment *L_k_* and the feed velocity *F_k_*, written as follows:

(6) $$T_{v,k}=\frac{L_{k}}{F_{k}}=\frac{\left\|P_{k+1}-P_{k}\right\|}{F_{k}}$$

**Figure 3:**
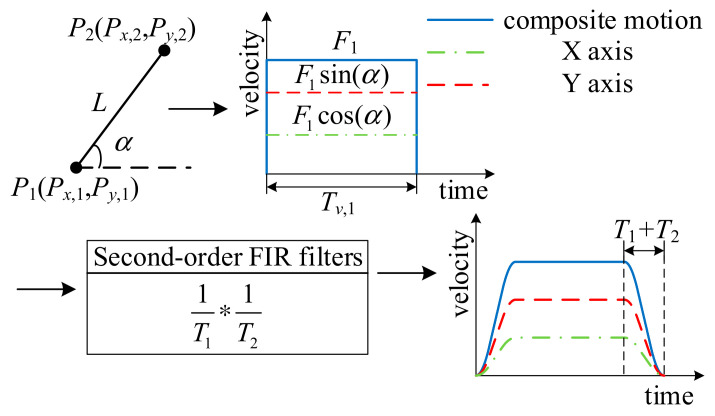
Velocity curve based on FIR filtering.

As shown in Figure 3, the angle between the line segment *P*_1_*P*_2_ and the horizontal direction is *α*. The speed pulses of the X-axis and the Y-axis are the projection of the speed pulse *F*_1_ of the synthetic motion in the X-axis and Y-axis directions. When the obtained speed pulse is filtered twice with FIR filters with delay times of *T*_1_ and *T*_2_, it is possible to generate an S-shaped velocity curve which is frequently observed in velocity planning. The velocity equation obtained for the condition *T_v_*_,*k*_ > *T*_1_ + *T*_2_ shows that the maximum acceleration *a*_max_ and jerk *j*_max_ are calculated as follows:

(7) $$\left\{\begin{array}{c} a_{\max}=\frac{F_{k}}{T_{1}}\\ j_{\max}=\frac{F_{k}}{T_{1}T_{2}} \end{array}\right.$$

**Figure 4:**
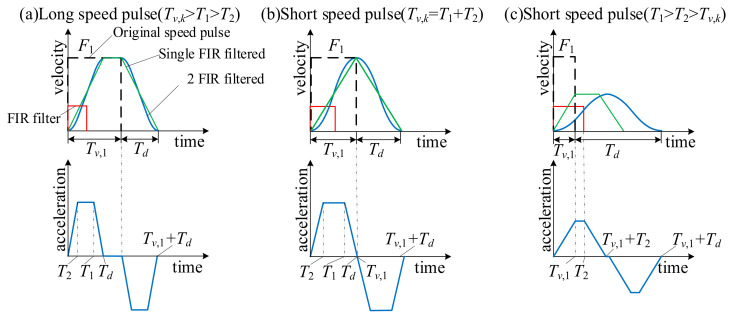
Velocity and acceleration curve of twice filtering. (**a**) Long speed pulse (*T_v_*_,*k*_ > *T*_1_ + *T*_2_). (**b**) Short speed pulse (*T_v_*_,*k*_ = *T*_1_ + *T*_2_) (**c**) Short speed pulse (*T_v_*_,*k*_ < *T*_1_ < *T*_2_).

To ensure that the acceleration curve is continuous after twice filtering, the condition *T*_1_ > *T*_2_ needs to be satisfied [24]. There are three different situations when a velocity pulse is filtered twice, which are divided into *T_v_*_,*k*_ > *T*_1_ + *T*_2_, *T_v_*_,*k*_ = *T*_1_ + *T*_2_, and *T_v_*_,*k*_ < *T*_1_ < *T*_2_ according to the quantitative relationship between the processing time of the servo axis and the delay time of the filter. As shown in Figure 4, Figure 4a displays a long speed pulse, while Figure 4b,c illustrate short speed pulses. Figure 4a shows that when the processing time of the servo axis satisfies *T_v_*_,*k*_ > *T*_1_ + *T*_2_, a smooth velocity curve can be generated, and the acceleration curve is also trapezoidal. In Figure 4a, it can be concluded that there are three stages in the filtered speed, which are the acceleration stage, the constant speed stage, and the deceleration stage. As shown in Figure 4b, under the condition that the processing time of the servo axis satisfies *T_v_*_,*k*_ = *T*_1_ + *T*_2_, there is no constant velocity stage in the velocity and acceleration curves, only acceleration and deceleration stages, and the processing time of the servo axis of the velocity and acceleration curves is short. As can be observed from Figure 4c, when the processing time of the servo axis satisfies *T_v_*_,*k*_ < *T*_1_ < *T*_2_, the filtered velocity and acceleration cannot reach the maximum limit values, but a smooth velocity curve can still be generated. In the three situations mentioned above, the total processing time is determined as follows:

(8) $$T_{v,k}+T_{d}=T_{v}+T_{1}+T_{2},\mathrm{where}\;T_{d}=T_{1}+T_{2}$$

where *T_d_* is total delay time produced by filtering twice.

Considering that in actual processing, such as automobile mold test samples, contain various complex path characteristics, the G-code usually consists of a mixture of long line segments and micro line segments, or only a pattern composed of spline curves is given, it is necessary to sample points for the pattern. In order to ensure the universality of the proposed method, this paper initially pre-discretizes the target pattern into multiple micro line segments. On this basis, we propose a velocity adjustment scaling factor *φ_v_* for controlling the global blending error of multiple micro segments in the motion planning of the servo platform. After obtaining the interpolation point of the servo platform over time, the real-time position of the galvanometer is determined by vector subtraction.

### 2.4. Pre-Discretization Algorithm for Initial Patterns

The first step of the proposed method is to pre-discretize the initial pattern or the initial G-code. For the former, the whole pattern needs to be divided into multiple micro line segments according to certain rules, and for the latter, the long G-code must be decomposed into micro line segments. Finally, the dense discrete micro line segments are utilized as the input of the algorithm to carry out velocity planning and motion distribution.

The precondition *T_v_*_,*k*_ < *T*_1_ < *T*_2_ and the critical condition *T_v_*_,*k*_ = *T*_2_ are the premises of the global blending error control algorithm for multiple micro line segments. At this point, the acceleration of the system reaches its maximum value, so the minimum time for discretization is *T_div_*_,min_ = *T*_2_. When the initial input is simply the whole pattern, the discretization of the initial path can be performed directly following *T_div_*_,min_. When the initial input is a mixture of long and micro G-codes, it is necessary to read the whole G-code first, marking the segment with the speed pulse width *T_v,k_* > *T_div_*_,min_ as a long line segment and discretizing this long line segment. To achieve the discretization of long line segments, we propose a scaling factor *k_s_* for pre-discretization, and the speed pulse width of the micro line segment is set to *T_v_*_,*s*_:

(9) $$T_{v,s}=k_{s}T_{2},k_{s}\in (0,1)$$

We suppose the total movement time of a long line segment with the starting point *P_k_*_-1_(*X_k_*_−1_,*Y_k_*_−1_) and the ending point *P_k_* (*X_k_*,*Y_k_*) is denoted as *T_k_*. Therefore, the number of micro line segments into which the long G-code is discretized is shown as follows:

(10) $$N=\mathrm{ceil}(\frac{T_{k}}{T_{\nu ,s}}),N\in N^{*}\mathrm{and}N\geq 2$$

The ceil function indicates upward rounding. Through the above discretization process, the long G-code can be linearly interpolated to obtain its middle new interpolation points *P_n_* = (*X_n_*,*Y_n_*). The coordinates of the new interpolation points in the X- and Y-axes are as follows where *n* = 1, 2, …, *N* − 1:

(11) $$\left\{\begin{array}{l} X_{n}=X_{k-1}+(X_{k}-X_{k-1})\frac{n}{N}\\ Y_{n}=Y_{k-1}+(Y_{k}-Y_{k-1})\frac{n}{N} \end{array}\right.$$

### 2.5. Global Blending Error Control and Motion Distribution Algorithms

After obtaining multiple consecutive micro line segments, from the discussion in Section 2.3, it follows that the system has *T_v_*_,*k*_ < *T*_2_ < *T*_1_ in this condition. Consequently, the max velocity and acceleration that can be achieved by the system at this point are calculated as follows:

(12) $$\left\{\begin{array}{c} \nu_{\max}=\frac{F_{k}T_{\nu ,k}}{T_{1}}\\ a_{\max}=\frac{F_{k}T_{\nu ,k}}{T_{1}T_{2}} \end{array}\right.$$

The S-shaped velocity profile obtained from the short speed pulse after filter FIR twice FIR is calculated as follows:

(13) $$\begin{array}{l} v(t)=x(t)*f_{1}(t)*f_{2}(t)\\ =\left\{\begin{array}{cc} \frac{1}{2}\frac{F_{k}}{T_{1}T_{2}}t^{2} & 0\leq t\leq T_{\nu ,k}\\ \frac{1}{2}\frac{F_{k}T_{\nu ,k}^{\;2}}{T_{1}T_{2}}+\frac{F_{k}T_{\nu ,k}}{T_{1}T_{2}}(t-T_{\nu ,k}) & T_{\nu ,k}\leq t\leq T_{2}\\ \frac{F_{k}T_{\nu ,k}}{T_{1}}-\frac{1}{2}\frac{F_{k}}{T_{1}T_{2}}{\left(T_{2}+T_{\nu ,k}-t\right)}^{2} & T_{2}\leq t\leq T_{2}+T_{\nu ,k}\\ \frac{F_{k}T_{\nu ,k}}{T_{1}} & T_{2}+T_{\nu ,k}\leq t\leq T_{1}\\ \frac{F_{k}T_{\nu ,k}}{T_{1}}-\frac{1}{2}\frac{F_{k}}{T_{1}T_{2}}{\left(t-T_{1}\right)}^{2} & T_{1}\leq t\leq T_{1}+T_{\nu ,k}\\ \frac{F_{k}T_{\nu ,k}}{T_{1}}-\frac{1}{2}\frac{F_{k}T_{\nu ,k}^{\;2}}{T_{1}T_{2}}-\frac{F_{k}T_{\nu ,k}}{T_{1}T_{2}}(t-(T_{1}+T_{\nu ,k})) & T_{1}+T_{\nu ,k}\leq t\leq T_{1}+T_{2}\\ \frac{1}{2}\frac{F_{k}}{T_{1}T_{2}}{\left(T_{1}+T_{2}+T_{\nu ,k}-t\right)}^{2} & T_{1}+T_{2}\leq t\leq T_{1}+T_{2}+T_{\nu ,k} \end{array}\right. \end{array}$$

The key point of the global blending error control algorithm proposed in this paper for multiple micro segments is the derivation of a theoretical formula for the global error *ε_k_*, as shown in Equation (16). It can be seen that the error is related to both the width and the height of the initial velocity pulse. Based on this, a velocity adjustment scaling factor *ϕ_v_* is proposed, which can be calculated by Equation (21). The global error is constrained by adjusting the initial velocity pulse by *ϕ_v_*.

**Figure 5:**
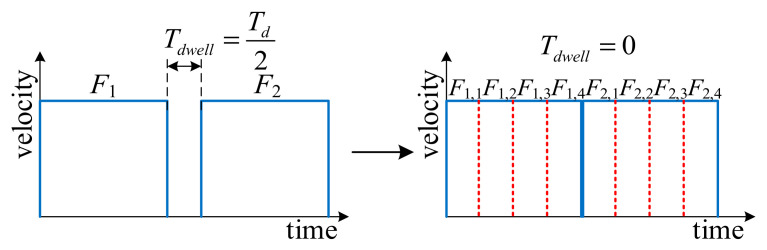
Motion overlap of multiple micro segments.

The preceding authors proposed a motion planning algorithm for macro–micro structure based on FIR filter [20], which is only applicable to patterns composed of consecutive long segments. In this algorithm, a dwell time is inserted between two adjacent long speed pulses, denoted as *T_dwell_*. When *T_dwell_* = *T_d_*, an accurate point-to-point motion is obtained, and when *T_dwell_* < *T_d_*, the overall running time is reduced, but it results in the introduction of contouring errors. On this basis, the cornering error is constrained to a specified range through the modification of the dwell time *T_dwell_* situated between two adjacent speed pulses, where the minimum value of *T_dwell_* is *T_d_*/2. The proposed method modifies the dwell time *T_dwell_* such that *T_dwell_* = 0 between adjacent speed pulses. As shown in Figure 5, each long segment is discretized into four micro segments, and each long speed pulse *F_k_* is similarly discretized into four adjacent short speed pulses *F_k_*_,*n*_, *n* = 1, 2, 3, 4, where n is the number of short segments into which the long segment is discretized. The discrete short speed pulses are connected end to end, further optimizing the overall running time, and the global blending error is also derived in the following for this condition.

**Figure 6:**
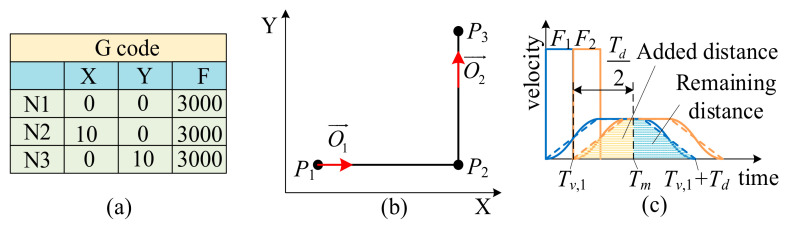
Contour error of two adjacent micro segments. (**a**) Initial G-code. (**b**) Target trajectory of adjacent micro segments. (**c**) Filtered velocity blending schematic diagram.

In the case of two adjacent micro segments, as shown in Figure 6a, line N1 represents the starting point *P*_1_(*P_x_*_,1_,*P_y_*_,1_), line N2 represents the first G01 code moving to the middle point *P*_2_(*P_x_*_,2_,*P_y_*_,2_), and line N3 represents the second G01 code moving to the ending point *P*_3_(*P_x_*_,3_,*P_y_*_,3_). As shown in Figure 6b, the unit vector of the *P*_1_*P*_2_ segment is $\vec{O_{1}}$ and the unit vector of the *P*_2_*P*_3_ segment is $\vec{O_{2}}$, and assuming that the feed velocity of both G-codes are equal, i.e., *F*_1_ = *F*_2_ = *F*_3_, the durations of the short speed pulses of the two segments are also equal, i.e., *T_v_*_,1_ = *T_v_*_,2_. At this time, the maximum contour error *ε* of the system occurs at the angular bisector of ∠*P*_1_*P*_2_*P*_3_, and the moment is denoted as the maximum error time *T_m_*, which is the delay *T_d_*/_2_ at the beginning of the second G-code, that is, *T_m_* = *T_v_*_,1_ + *T_d_*/2.

The contour error is a consequence of the fact that, when the first G-code runs to *T_v_*_,1_, the second G-code starts to run, resulting in a change in the feed direction. In order to facilitate the calculation of the maximum contour error *ε*, the S-shaped velocity profile of Equation (13) is approximated as a trapezoidal velocity profile as in the following Equation (14):

(14) $$\nu (t)=\left\{\begin{array}{cc} \frac{F_{k}T_{\nu ,k}}{T_{1}(T_{2}+T_{\nu ,k})}t & 0\leq t\leq T_{2}+T_{\nu ,k}\\ \frac{F_{k}T_{\nu ,k}}{T_{1}} & T_{2}+T_{\nu ,k}\leq t\leq T_{1}\\ \frac{F_{k}T_{\nu }}{T_{1}}-\frac{F_{k}T_{\nu ,k}}{T_{1}(T_{2}+T_{\nu ,k})}(t-T_{1}) & T_{1}\leq t\leq T_{1}+T_{2}+T_{\nu ,k} \end{array}\right.$$

As shown in Figure 6c, the maximum contour error occurs at *T_m_*. The first G-code gradually starts to slow down from *T_m_*, and this distance is called “remaining distance”, denoted by *l_r_*, which corresponds to the blue area in the figure. Meanwhile, the distance from the beginning of the second G-code to *T_m_* is called “added distance” and is denoted by *l_a_*, which corresponds to the red area in the figure. Equation (15) is obtained by analyzing the integration of the trapezoidal velocity curve at *T_m_*, written as follows:

(15) $$\begin{array}{l} l_{r}=\int_{T_{v,1}+\frac{T_{d}}{2}}^{T_{v,1}+T_{d}}{v1}^{\prime }d\tau =\left\{\begin{array}{cc} \frac{F_{1}T_{\nu ,1}}{2T_{1}}\left(T_{1}-T_{\nu ,1}\right) & T_{2}+T_{\nu ,1}\leq \frac{T_{1}+T_{2}}{2}\\ \frac{F_{1}T_{\nu ,1}}{2T_{1}\left(T_{2}+T_{\nu ,1}\right)}{\left(\frac{T_{1}+T_{2}}{2}\right)}^{2} & T_{2}+T_{\nu ,1}\geq \frac{T_{1}+T_{2}}{2} \end{array}\right.\\ l_{a}=\int_{0}^{\frac{T_{d}}{2}}{v2}^{\prime }d\tau =\left\{\begin{array}{cc} \frac{F_{2}T_{\nu ,2}}{2T_{1}}\left(T_{1}-T_{\nu ,2}\right) & T_{2}+T_{\nu ,2}\leq \frac{T_{1}+T_{2}}{2}\\ \frac{F_{2}T_{\nu ,2}}{2T_{1}\left(T_{2}+T_{\nu ,2}\right)}{\left(\frac{T_{1}+T_{2}}{2}\right)}^{2} & T_{2}+T_{\nu ,2}\geq \frac{T_{1}+T_{2}}{2} \end{array}\right. \end{array}$$

The maximum contour error *ε* can be expressed as the following Equation (16):

(16) $$\varepsilon =∥l_{r}O_{2}-l_{a}O_{1}∥=\sqrt{l_{r}^{2}+l_{a}^{2}-2l_{r}l_{a}(O_{1}\cdot O_{2})}$$

**Figure 7:**
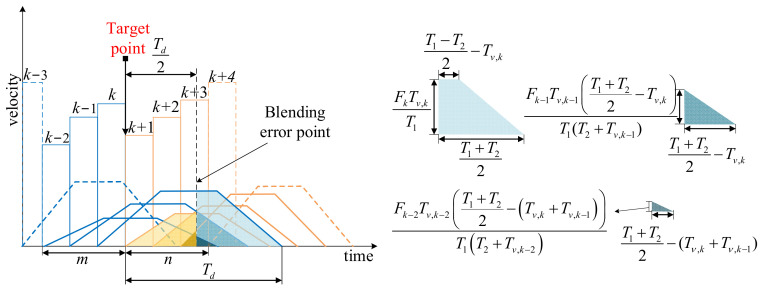
Global blending error of multiple micro segments.

For the continuous multiple micro segments trajectory, as shown in Figure 7, the kth contour point generates the largest contour error at *T_m_*_,*k*_, as shown in the previous section. The endpoint of this speed pulse is called the target point, and extending the target point backward by *T_d_*/2 gives the point where the maximum contour error occurs, i.e., *T_m,k_* = *T_v_*_,*k*_ + *T_d_*/2. The global blending error here is affected by the remaining distance of the front *m* speed pulses and the added distance of the rear *n* speed pulses, with each remaining and added distance influencing the mixing error along its feed direction **O***_k_*. The maximum error of the segment is expressed as follows:

(17) $$\varepsilon_{k}=∥\sum_{i=0}^{n-1}\vec{l_{k+i}}-\sum_{j=0}^{m-1}\vec{l_{k-j-1}}∥=∥\sum_{i=0}^{n-1}l_{k+i}O_{k+i}-\sum_{j=0}^{m-1}l_{k-j-1}O_{k-j+1}∥$$

where *l_k_*_−*j*−1_ denotes the remaining distance generated by the front *m* speed pulses, *j* = 1, 2, …, *m*; and *l_k+i_* denotes the added distance generated by the rear *n* speed pulses, *i =* 1, 2, …, *n*.

The remaining distance *l_k_*_−*j*−1_ can be calculated by computing the integral of each speed pulse as follows:

(18) $$l_{k-j+1}=\int_{t=\sum_{p=1}^{k}T_{\nu ,p}+\frac{T_{d}}{2}}^{t=\sum_{p=1}^{k}T_{\nu ,p}+T_{d}}\nu_{k-j+1}^{\prime }dt,j=1,2,\ldots ,m$$

where *T_v,p_* denotes the duration of the *p*th speed pulse, *p =* 1, 2, *…*, *k*. Figure 7 illustrates that the integral velocity profile computation can be divided into two geometries: trapezoidal and triangular. The first type is that the intersecting part is trapezoidal, with the height of the trapezoid representing the peak velocity of the trapezoidal velocity curve. The second type is that the intersecting part is triangular. *l_k_*_−*j*−1_ can be expressed as follows:

(19) $$l_{k-j+1}=\left\{\begin{array}{cc} \frac{F_{k-j+1}T_{\nu ,k-j+1}}{2T_{1}}\left(T_{1}-T_{\nu ,k-j+1}-2\sum_{k_{1}=k-j+2}^{k}T_{\nu ,k_{1}}\right)\; & \mathrm{if}\;\sum_{k_{1}=k-j+1}^{k}T_{\nu ,k_{1}}\leq \frac{T_{1}-T_{2}}{2}\\ \frac{F_{k-j+1}T_{\nu ,k-j+1}}{2T_{1}\left(T_{2}+T_{\nu ,k-j+1}\right)}{\left(\frac{T_{1}+T_{2}}{2}-\sum_{k_{1}=k-j+2}^{k}T_{\nu ,k_{1}}\right)}^{2} & \mathrm{if}\;\sum_{k_{1}=k-j+1}^{k}T_{\nu ,k_{1}}\geq \frac{T_{1}-T_{2}}{2} \end{array}\right.,j=1,2,\ldots ,m$$

Similarly, the added distance *l_k+i_* due to the *n* speed pulses after the *k*th contour point can be expressed as follows:

(20) $$l_{k+i}=\int_{t=\sum_{p=1}^{k}T_{\nu ,p}}^{t=\sum_{p=1}^{k}T_{\nu ,p}+\frac{T_{d}}{2}}\nu_{k+i}^{\prime }dt,i=1,2,\ldots ,n$$

It can be seen from Figure 7 that there are two types of their geometries, and *l_k+i_* can be expressed as follows:

(21) $$l_{k+i}=\left\{\begin{array}{cc} \frac{F_{k+i}T_{\nu ,k+i}}{2T_{1}}\left(T_{1}-T_{\nu ,k+i}-2\sum_{k_{2}=k+1}^{k}T_{\nu ,k_{2}}\right)\; & \mathrm{if}\;\sum_{k_{2}=k+1}^{k}T_{\nu ,k_{2}}\leq \frac{T_{1}-T_{2}}{2}\\ \frac{F_{k+i}T_{\nu ,k+i}}{2T_{1}\left(T_{2}+T_{\nu ,k+i}\right)}{\left(\frac{T_{1}+T_{2}}{2}-\sum_{k_{2}=k+1}^{k}T_{\nu ,k_{2}}\right)}^{2} & \mathrm{if}\;\sum_{k_{2}=k+1}^{k}T_{\nu ,k_{2}}\geq \frac{T_{1}-T_{2}}{2} \end{array}\right.,i=1,2,\ldots ,n$$

The global blending error *ε_k_* at the *k*th corner can be obtained by substituting Equation (19) and Equation (21) into Equation (17). In order to control the global error at this location within the constraints, we propose a velocity adjustment scaling factor *ϕ_v_*_,*k*_ for adjusting the initial speed impulse, which can be expressed as follows:

(22) $$\varphi_{v,k}=\frac{\varepsilon_{\mathrm{allow}}}{\varepsilon_{k}}$$

where *ε*_allow_ is the constraint value of the servo platform contour error, here we take the FOV range of the galvanometer as its constraint. Therefore, the velocity adjustment scaling factor at the *k*th corner affected by *m* + *n* speed pulses is *ϕ_v_*_,*k*_, subject to the contour error constraints.

Since each speed pulse affects the blending error at more than one corner and the global blending error at each corner is not equal, multiple velocity adjustment scaling factors are obtained for a single speed pulse. To ensure that the blending error at each corner satisfies the constraint, the minimum of the multiple velocity adjustment scaling factors at each speed pulse is used as the final velocity adjustment scaling factor *ϕ_v_* for all speed pulses.

(23) $$\varphi_{v}=\min\left\{\varphi_{v,1},\varphi_{v,2},\ldots ,\varphi_{v,E-1}\right\}$$

where *ϕ_v_*_,*E*−1_ denotes the scaling factor for the last segment of the velocity pulse. The feed velocity and duration corresponding to the speed pulse are adjusted as follows:

(24) $$\left\{\begin{array}{c} {F_{k}}^{\prime }=\varphi_{v}F_{k}\\ {T_{v,k}}^{\prime }=\frac{T_{v,k}}{\varphi_{v}} \end{array}\right.,k=1,2,\ldots ,E-1,E\in N^{*}$$

where ${F_{k}}^{\prime }$ and ${T_{v,k}}^{\prime }$ are the adjusted feed velocity and duration, and *E* denotes the number of contour points of the processed pattern.

After obtaining the kinematic curve of the motion platform under the error constraint, its motion trajectory is interpolated according to the interpolation time *t_c_* to obtain the displacement vector $\vec{p_{p,i}}$ of the servo platform, *i* = 1, 2, …, *m*, where *m* denotes the number of displacement vectors after interpolation. Since the motion of the servo platform and galvanometer are coupled, it is essential to ensure the synchronous operation of the servo platform and galvanometer; the following relationship should also be satisfied in the parameter of time:

(25) $$\frac{\left|\vec{p_{p,i}}\right|}{v_{p,i}}=\frac{\left|\vec{p_{g,i}}\right|}{v_{g,i}}=\frac{\left|\vec{p_{t,i}}\right|}{v_{t,i}}$$

where $\vec{p_{t,i}}$ is the displacement vector of the target trajectory, *i =* 1, 2, …, *m*, and *v_p_*_,*i*_, *v_g_*_,*i*_, *v_t_*_,*i*_ are the velocities of the servo platform, the galvanometer, and the target trajectory.

**Figure 8:**
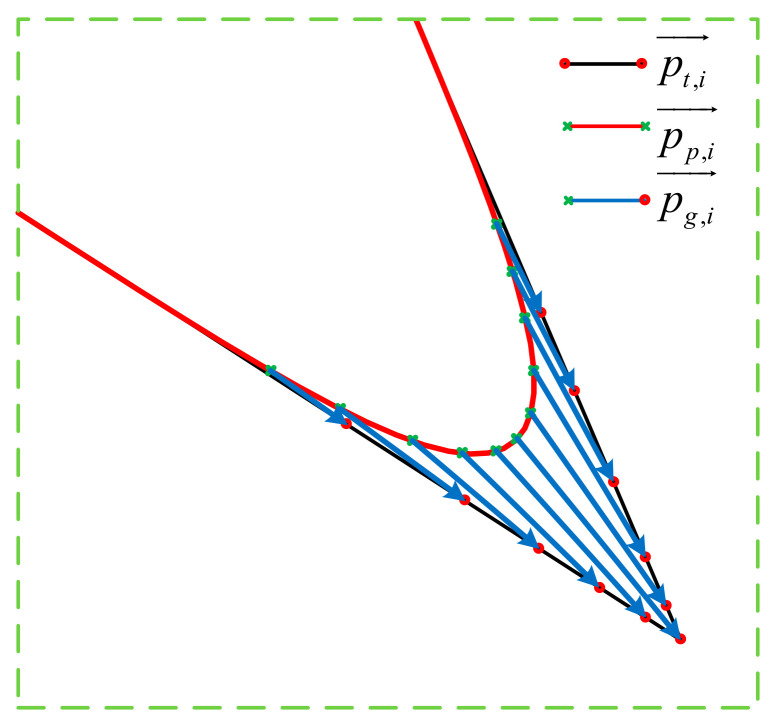
Equal scale mapping.

In order to satisfy the above conditions, under the premise that the servo platform displacement vector and the total length of the target trajectory are known, we resample the target trajectory using the method of equal scale mapping. The relationship between the target trajectory displacement vector after equal scale mapping, the galvo displacement vector, and the servo platform displacement vector is shown in Figure 8. There is a deceleration process at the corner; the velocity is minimum at the corner, so in the same interpolation period, *t_c_*, $\vec{p_{p,i}}$ becomes shorter as it approaches the corner. Multiplying the ratio of the individual servo platform displacement vector to the total length of the servo platform motion trajectory with the total length of the target trajectory further obtains the displacement vector of the target trajectory, which can be expressed as follows:

(26) $$\frac{\left|\vec{p_{p,i}}\right|}{\sum_{i=1}^{m}\left|\vec{p_{p,i}}\right|}=\frac{\left|\vec{p_{t,i}}\right|}{\sum_{i=1}^{m}\left|\vec{p_{t,i}}\right|}$$

The relationship between the displacement vectors of the servo platform, the galvanometer, and the target trajectory is shown by Equation (3). After obtaining the displacement vector $\vec{p_{t,i}}$ of the target trajectory, we calculate the trajectory $\vec{p_{g,i}}$ of the galvanometer according to the vector subtraction, *i* = 1, 2, …, *m*, which can be expressed as follows:

(27) $$\vec{p_{g,i}}=\vec{p_{t,i}}-\vec{p_{p,i}}$$

Based on the above algorithm, we obtain the position of the servo platform at each moment under the constraint of the FOV of the galvanometer and the position of the galvanometer at each moment. This enables us to perform the motion planning and trajectory distribution of macro–micro structure, and each parameter in the algorithm is derived theoretically and does not need to be determined by repeated experiments.

## 3. Simulation and Experimental Validation

A butterfly-shaped pattern is utilized for simulation verification and machining operations in order to confirm the viability of the proposed approach on the complex target pattern. This section compares three processing methods: the servo platform processing only (SPO), the previous linkage processing method (PLP) [18], and the proposed method in this paper. For a reasonable comparison, a conventional seven-stage acceleration and deceleration planning algorithm is used for point-to-point motion when processing with a servo platform only. Table 1 summarizes kinematic limits and key trajectory parameters while setting the FOV of the galvanometer to ±7 mm.

### 3.1. Simulation Results and Discussion

**Figure 9:**
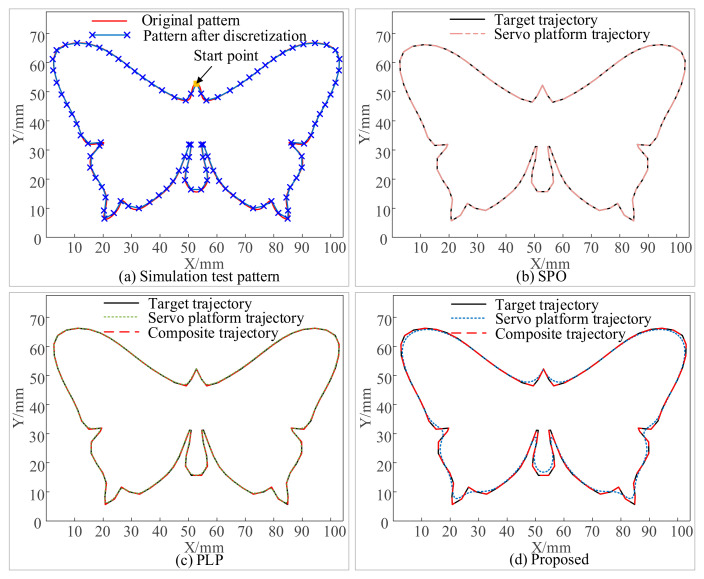
Simulation test and comparison. (**a**) Target pattern. (**b**–**d**) Trajectory distribution of SPO, PLP and proposed, respectively.

All algorithms are written in Matlab and run on a PC with an Intel(R) Core(TM) i5-12500@3.00 GHz and 16 GB RAM. As seen in Figure 9a, the test pattern has a height of 70 mm and a width of 100 mm. Following pre-discretization, there are 100 micro segments in total.

**Figure 10:**
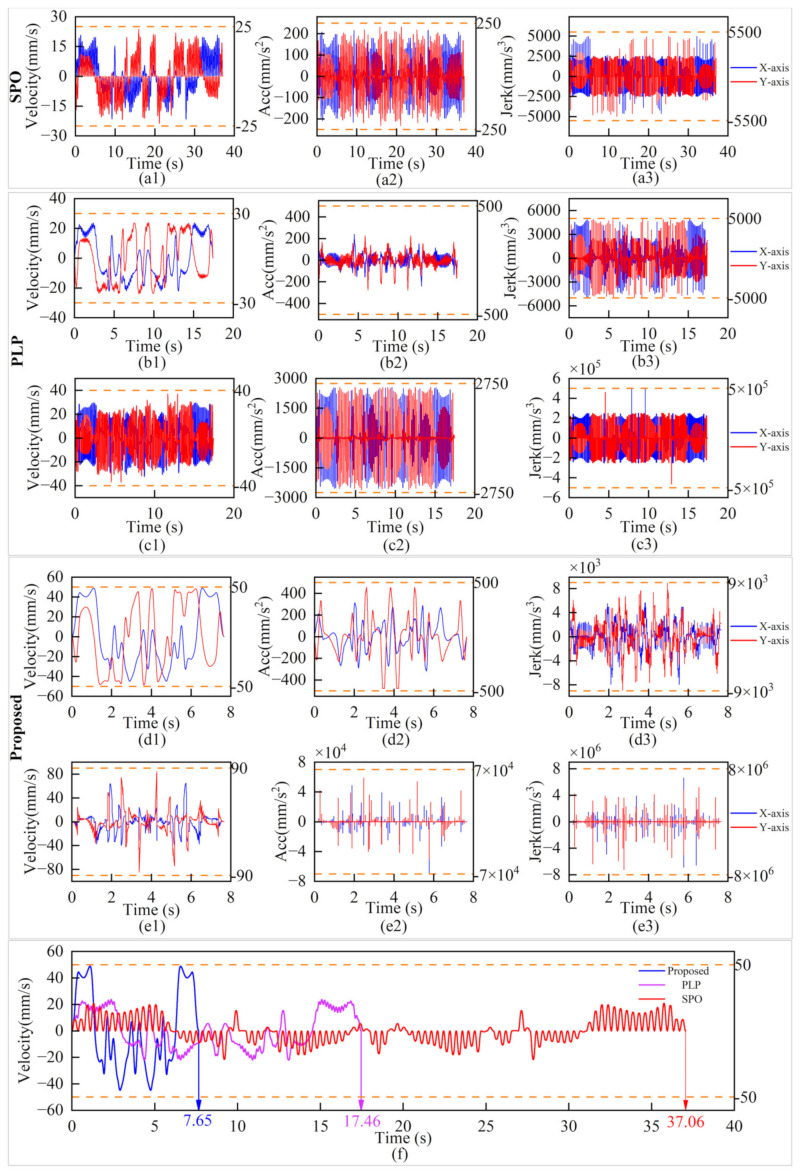
Simulation results for butterfly-shaped trajectory. (**a1**–**a3**) Velocity, acceleration, and jerk of servo platform with SPO. (**b1**–**b3**) Velocity, acceleration, and jerk of servo platform with PLP. (**c1**–**c3**) Velocity, acceleration, and jerk of galvanometer with PLP. (**d1**–**d3**) Velocity, acceleration, and jerk of servo platform with the proposed. (**e1**–**e3**) Velocity, acceleration, and jerk of galvanometer with the proposed. (**f**) Servo platform velocity along X-axis of SOP, PLP, and the proposed, respectively.

**Figure 11:**
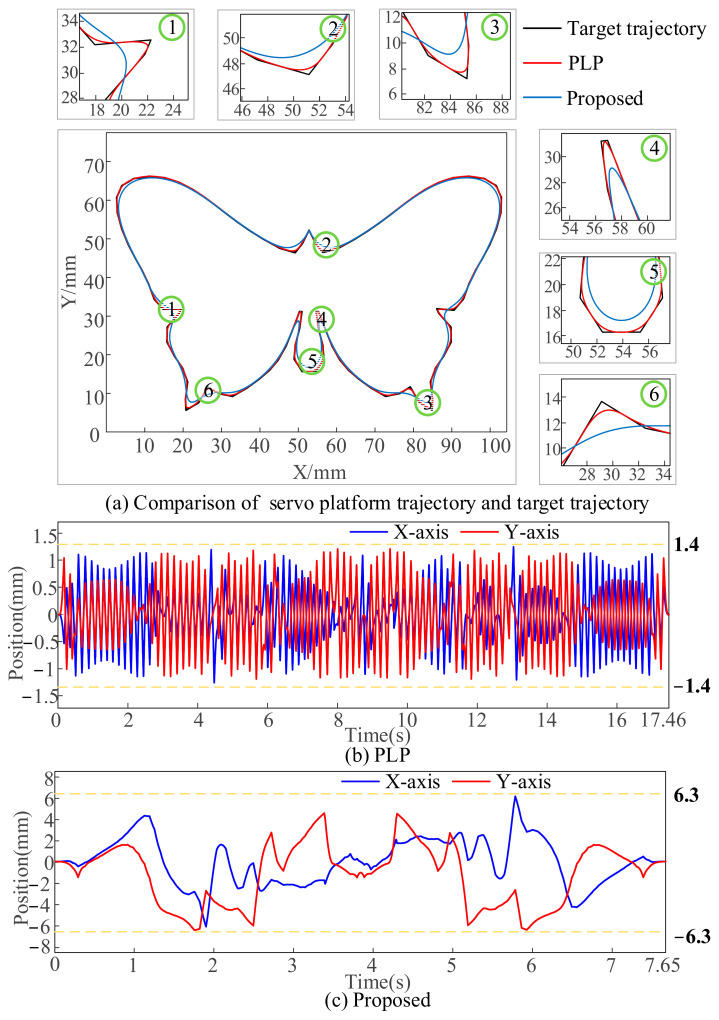
Comparison of PLP and the proposed. (**a**) Servo platform trajectory of PLP and the proposed. (**b**,**c**) Position of galvanometer’s axes of PLP and the proposed, respectively.

The simulation results are shown in Figure 9, Figure 10 and Figure 11. Figure 9b–d show the trajectory distributions of the SPO, PLP, and the proposed method, respectively. It can be seen that the trajectories of the composite motion and the target trajectory are completely overlapped, which indicates that none of the three methods introduces contouring errors. Figure 10(a1,a2,a3) show the velocity, acceleration and jerk profiles using SPO. In order to realize point-to-point motion, each axis of the servo platform needs to be accelerated from zero and decelerated to zero at each velocity pulse command, which extends the overall machining time. Figure 10b,c show the kinematic profiles using the PLP method. This method plans the composite motion and the servo platform motion separately and then subtracts the two to obtain the galvanometer motion. The total machining time of the method depends on the motion time of the servo platform, and as mentioned in Section 2.5, this method is not applicable for the motion planning of the servo platform in the case of multiple micro segments.

As shown in Figure 10(b1), the maximum velocity of the two axes of the servo platform under the PLP method is 30 mm/s, while the maximum velocity set by the simulation is 50 mm/s, which demonstrates that the method does not achieve the maximum velocity when dealing with the pattern of multiple micro segments. Meanwhile, its motion overlap time is small in comparison with that of the proposed method, which further prolongs the machining time. Figure 10d,e show the motion profiles of each axis of the servo platform and the galvanometer obtained using the proposed method. As shown in Figure 10(d1), the overall running velocity of the servo axis is very smooth, avoiding unnecessary acceleration and deceleration processes, and the maximum value can reach 50 mm/s. At the same time, the acceleration of the servo axis is within ±500mm/s^2^ and the jerk is within ±9 × 10^3^ mm/s^3^, which is within the set range. Comparing Figure 10(c1,c2) and Figure 10(e1,e2), it can be seen that the maximum value of the velocity of the galvanometer of the PLP method is 40 mm/s, and the maximum value of the acceleration is 2750 mm/s^2^, and the maximum value of the velocity of the galvanometer of the present method is 90 mm/s, and the maximum value of the acceleration is 7 × 10^4^ mm/s^2^. The velocity, acceleration, and jerk of the galvanometer of the proposed method are all within the set range, and the performance of the galvanometer is more fully utilized than that of the PLP method. This conclusion can also be demonstrated in Figure 11, which mainly compares the motion of the galvanometer using PLP and the proposed method.

As shown in Figure 11a, it can be seen that the motion trajectory of the servo platform with PLP is very close to the target trajectory, whereas the proposed method reduces the motion of the servo platform and increases the contour error of the servo stage trajectory under the constraint of FOV. This means that more motion is assigned to the galvanometer under the FOV constraint, as shown in Figure 11b,c. Figure 11b is the time–position plot of the galvanometer motion with the PLP, which can be seen as the movement only within ±1.4 mm, and Figure 11c is the time–position plot of the galvanometer motion with the proposed method, in which the motion range of the galvanometer is within ±6.3 mm.

The comparison of processing time and efficiency is shown in Figure 10f. The SPO takes about 37.06 s to complete the task, the PLP takes about 17.46 s to complete the task, and the proposed method takes only 7.65 s. It can be concluded that the proposed method can accurately and efficiently process the target trajectory without introducing contour error, and the efficiency is 79% higher than that of the SOP and 55% higher than that of the PLP.

### 3.2. Experimental Results

**Figure 12:**
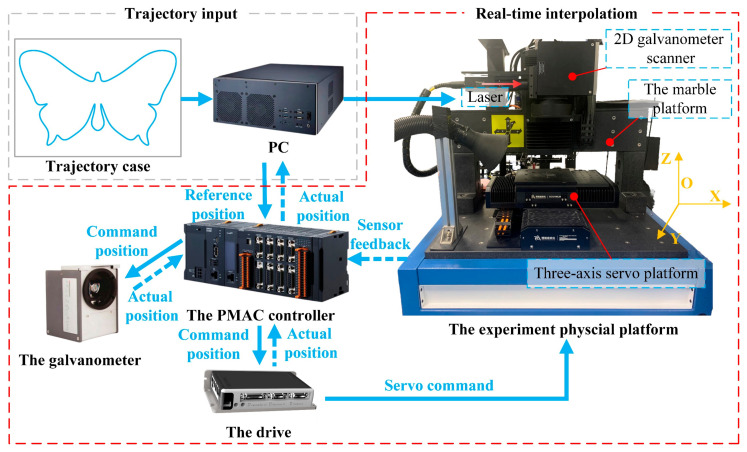
Configuration of the experimental physical platform setup.

To confirm the efficacy of the suggested linkage machining technique, an experimental setup including a 2D galvanometer, a three-axis servo platform, a marble stage, and a laser was constructed. As shown in Figure 12 for the constructed experimental platform, the laser processing software on the PC can realize the drawing and inputting of the target pattern, as well as the setting of some laser processing parameters. After receiving the trajectory data, the programmable multi-axis controller (PMAC) performs pre-discretization of the initial trajectory, motion planning, and trajectory distribution, and assigns interpolation points to the galvanometer and servo platform. In addition, the controller obtains the real-time feedback position signals through the position sensors on the servo platform and the galvanometer motor and transmits them to the PC. The structure of the experimental platform is shown below:

1. The PC is connected to the controller through a network cable to realize human–computer interaction and the sending of commands.
2. The controller model is PMAC-CK3M from OMRON (Osaka, Japan), which is mainly responsible for the operation of the algorithm, realizing the planning and decomposition of the trajectory, and sending corresponding control signals to the servo stage and the galvanometer.
3. The three-axis servo platform consists of three servo axes (X–Y–Z) and a marble stage with SERVOTRONIX model PRHD2 drives (Servotronix, Petah Tikva, Israel).
4. The 2D galvanometer is SCANLAB’s intelliSCAN14 (Scanlab GmbH, Puchheim, Germany), and the communication protocol is SL2-100.

**Figure 13:**
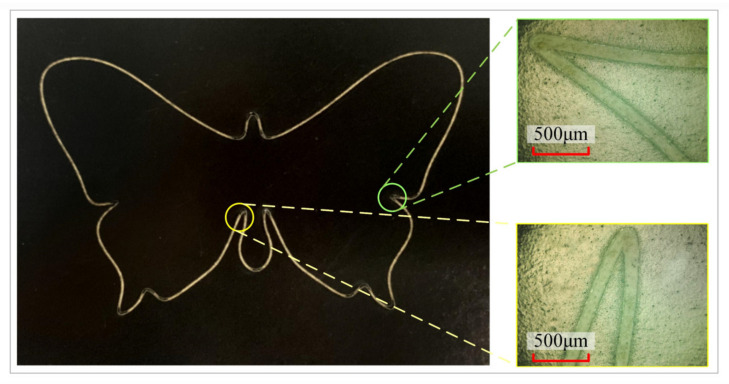
Processing result diagram of the butterfly pattern and locally magnified diagram at the corner.

Realistic machining experiments are performed on the butterfly-shaped pattern from the simulation in Section 3.1. The pattern is input into a PC, and the trajectory planning and decomposition are performed by the controller, which transmits the position commands to the galvanometer and the servo platform. In the experiment, a pulsed fiber laser with a center wavelength of 1064 nm is utilized on a black photo paper, and the processing results are shown in Figure 13.

**Figure 14:**
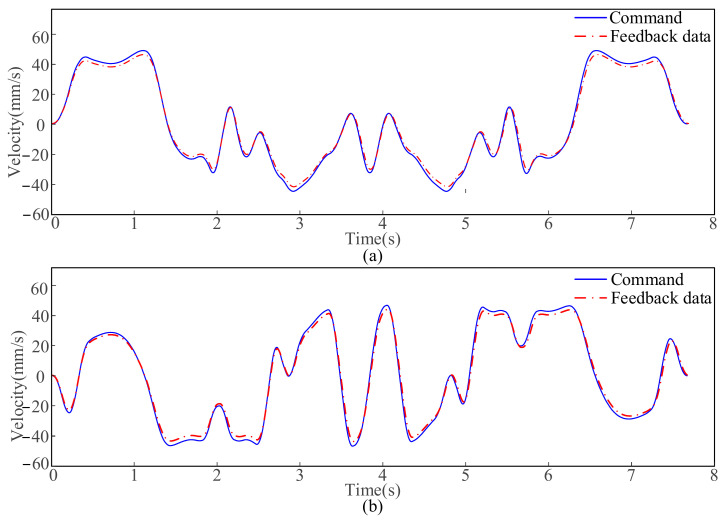
Comparison between feedback velocity and commanded velocity: (**a**) Velocity of X-axis; (**b**) Velocity of Y-axis.

Figure 13 depicts the processing results and local magnification, which shows that the trajectory at the sharp corners is smoother and free of distortion, ensuring the processing quality. As shown in Figure 14a,b, the velocity profiles of the servo axes in actual machining are compared with the input velocity profiles, from which it can be concluded that the velocity profiles planned by the proposed method can be practically utilized on the experimental platform and exhibit good tracking performance.

## 4. Conclusions

In this paper, the 2D galvanometer and servo platform are combined for linkage laser processing, which tackles the lack of separate movements of the two. Based on this macro–micro structure, a new method of laser linkage processing applicable to multiple micro-line segment paths is proposed. In order to improve the generalizability of the method, the method pre-discretizes the initial trajectory and discretizes the long line segments in the target trajectory into micro segments. The galvanometer FOV is used as a global mixing error constraint during macro structure planning, and the servo platform motion trajectory with bounded additive acceleration and smooth acceleration is generated by an FIR filter. A velocity adjustment scale factor is proposed to adjust the initial velocity pulse so that the contour error is within the constraint, which not only ensures the accuracy of the linkage machining but also fully utilizes the high dynamic performance of the galvanometer. After obtaining the motion trajectory of the servo platform, the real-time motion interpolation of the galvanometer is obtained by equal scale mapping and vector subtraction.

The proposed method generates the advantages of high efficiency and high applicability. The target trajectory is set as a butterfly-shaped pattern, and the effectiveness and feasibility of the proposed method for motion planning and decomposition of trajectory composed of multiple micro segments are verified by simulation and experiment. Simulation and experimental results show that the total processing time of the method is reduced by 79% compared with servo platform motion only and 55% compared with the previous linkage processing method, while the proposed method can be practically applied to the laser linkage processing platform.

## Figures and Tables

**Table 1:** Kinematic limits and key trajectory parameters.

Structure	Filter Time Delay	Max Velocity	Max Acceleration	Max Jerk
Servo platform	*T*_1, *p*_ = 200 ms, *T*_2,*p*_ = 100 ms	50 mm/s	2 × 10^4^ mm/s^2^	1 × 10^5^ mm/s^3^
Galvanometer	*T*_1, *g*_ = 20 ms, *T*_2,*g*_ = 10 ms	200 mm/s	2 × 10^5^ mm/s^2^	2 × 10^7^ mm/s^3^

## Data Availability

Data are contained within the article.

## Abbreviations

Variable Table	
*x_g_*, *y_g_*	displacements of the galvanometer in the X and Y directions
*x*_FOV_, *y*_FOV_	ranges of the FOV in the X and Y directions
*x_p_*, *y_p_*	displacements of the servo platform in the X and Y directions
$\vec{p_{t}}$	target displacement vector
$\vec{p_{p}}$	servo platform displacement vector
$\vec{p_{p}}$	galvanometer displacement vector
*T_i_*	delay time of the filter
*t_c_*	interpolation period
*f_i_*(*t*)	FIR filter
*v*(*t*)	filtered velocity sequence of synthetic motion
**P**_k_	series of Cartesian coordinates
*F_k_*	tangential feed pulse commands
*T_v_*_,*k*_	speed pulse width
*L_k_*	length of the line segment
*a*_max_,*j*_max_	maximum acceleration and jerk
*T_d_*	total delay time
*k_s_*	scaling factor
*T_v_*_,*s*_	speed pulse width of micro line segment
*X_n_,Y_n_*	the new interpolation points in the X- and Y-axes
*ϕ_v_*	velocity adjustment scaling factor
*T_dwell_*	dwell time between two adjacent long speed pulses
*F_k_*_,*n*_	short speed pulses
*T_m_*	moment of the maximum error time
*l_r_*	remaining distance
*l_a_*	added distance
*ε*	maximum contour error
$\vec{O_{i}}$	unit vector of the segment
*ε*_allow_	constraint value of the servo platform contour error
